# Age-Related Differences in Early Cortical Representations of Target Speech Masked by Either Steady-State Noise or Competing Speech

**DOI:** 10.3389/fpsyg.2022.935475

**Published:** 2022-08-04

**Authors:** Bruce A. Schneider, Cristina Rabaglia, Meital Avivi-Reich, Dena Krieger, Stephen R. Arnott, Claude Alain

**Affiliations:** ^1^Department of Psychology, Human Communication Laboratory, University of Toronto Mississauga, Mississauga, ON, Canada; ^2^Department of Communication Arts, Sciences, and Disorders, Brooklyn College, City University of New York, Brooklyn, NY, United States; ^3^Rotman Research Institute, Baycrest Centre, Toronto, ON, Canada; ^4^Department of Psychology, St. George Campus, University of Toronto, Toronto, ON, Canada

**Keywords:** aging, release from masking, ERP, word in noise, acoustic change complex

## Abstract

Word in noise identification is facilitated by acoustic differences between target and competing sounds and temporal separation between the onset of the masker and that of the target. Younger and older adults are able to take advantage of onset delay when the masker is dissimilar (Noise) to the target word, but only younger adults are able to do so when the masker is similar (Babble). We examined the neural underpinning of this age difference using cortical evoked responses to words masked by either Babble or Noise when the masker preceded the target word by 100 or 600 ms in younger and older adults, after adjusting the signal-to-noise ratios (SNRs) to equate behavioural performance across age groups and conditions. For the 100 ms onset delay, the word in noise elicited an acoustic change complex (ACC) response that was comparable in younger and older adults. For the 600 ms onset delay, the ACC was modulated by both masker type and age. In older adults, the ACC to a word in babble was not affected by the increase in onset delay whereas younger adults showed a benefit from longer delays. Hence, the age difference in sensitivity to temporal delay is indexed by early activity in the auditory cortex. These results are consistent with the hypothesis that an increase in onset delay improves stream segregation in younger adults in both noise and babble, but only in noise for older adults and that this change in stream segregation is evident in early cortical processes.

## Introduction

Communication in everyday life often requires listeners to navigate complex auditory scenes, full of competing information arriving at the listeners’ ears concurrently with the target message. Further, the challenging task of processing soundscapes becomes increasingly difficult as we age. Importantly, older adults exhibit difficulty comprehending speech when competing sounds are present (e.g., Anderson et al., 2018; Avivi-Reich et al., 2018)—holding a coherent conversation in a crowded restaurant with piped-in music, for example, might pose something akin to a herculean task for older adults, even if they possess clinically normal hearing for their age group (Humes, 2020).

In adverse listening situations, listeners must be able to isolate a reasonably veridical sensory representation of the target message, thus allowing further processing to take place. In order to do so, the auditory scene must be parsed into its auditory components (stream segregation, Bregman, 1990), thereby allowing listeners to focus their attention on the target signal. This can be a demanding task, requiring processing at both peripheral and central levels. Sound sources that temporally and spectrally overlap the target signal create excitation patterns in the cochlea and along the auditory nerve that overlap with those of the target signal. This type of interference often is referred to as energetic masking or peripheral masking (e.g., Durlach et al., 2003; Vander Werff et al., 2021). In addition, when the masker contains speech, it is likely to initiate lexical processing that could potentially allow irrelevant content to interfere with the processing of the target message at more central levels. This type of interference is referred to as informational masking (Freyman et al., 1999; Durlach et al., 2003; Schneider et al., 2007, 2010; Kidd et al., 2008; Jagadeesh and Uppunda, 2021), and is thought to affect higher more central processes than energetic masking (Arbogast et al., 2002; Freyman et al., 2004; Ihlefeld and Shinn-Cunningham, 2008; Szalárdy et al., 2019; Vander Werff et al., 2021).

Listeners can alleviate the interference cause by competing sound sources if they are able to segregate the incoming auditory input into separate auditory streams and correctly identify the target stream. Successful stream segregation, leading to a reduction in the interference caused by the maskers, is referred to as “release from masking” (e.g., Brungart et al., 2001; Durlach et al., 2003; King et al., 2020). The ability to do so depends on the perceptual similarities and dissimilarities between the target signal and the other competing sound sources present in the same auditory scene. Any differences among the sound sources could assist stream segregation, thereby providing a release from masking (Bregman, 1990). Different acoustic cues that could assist stream segregation have been previously investigated (e.g., Brungart et al., 2001; Humes et al., 2006; Vongpaisal and Pichora-Fuller, 2007; King et al., 2020; Rajasingam et al., 2021). These cues include acoustic dissimilarities between the target and masker/s (such as differences in F0 and spectrum) and temporal differences in the onset of successive sounds. Beyond these acoustic factors, knowledge-driven or top-down assisting cues, such as expectations, prior exposure and attention have also been found to affect stream segregation (e.g., Shinn-Cunningham and Best, 2008; Ragert et al., 2014).

Older adults, even those who are considered to have normal hearing for their age, show a reduced ability to use certain cues to enhance speech in noise perception (e.g., Dubno et al., 2002; Helfer and Freyman, 2008; Avivi-Reich et al., 2014; Stevenson et al., 2015; Roque et al., 2019). Importantly, all types of maskers do not have a similar effect on listeners across the lifespan. Maskers that seem particularly detrimental as one ages are those that contain competing speech (Tun and Wingfield, 1999; Helfer and Freyman, 2008; Rajan and Cainer, 2008). The disproportional difficulty older adults experience in multi-talker scenes compared with younger adults could be related to difficulties segregating the target stream from competing speech streams due to the acoustic similarity between them. While segregating a speech stream from noise streams, that contain no semantic information and significantly differ acoustically from the target, seems to be relatively automatic and less demanding (Snyder et al., 2006), segregating a target speech stream from other competing speech streams may require more attention and resources and result in less release from masking (Alain, 2004). In addition, it has been suggested that older adults benefit less from acoustic cues and perceptual opportunities, such as an onset delay between speech maskers and the target speech (Ben-David et al., 2012; Getzmann and Näätänen, 2015) compared to young adults. Considering these age-related findings, it is important to further examine how older adults differ from young adults in the ability to release speech from masking when attempting to identify word in noise from different types of maskers, and with different temporal relationships between maskers and target words.

In the present study, we focus on the degree of acoustic similarity between the target and competing auditory inputs, and the temporal cues derived from differences in sound onset. When the onset of a target sound and the onset of one or more competing auditory streams are separated in time, listeners take advantage of this temporal discrepancy to segregate a target sound within an auditory scene (Zwicker, 1965; McFadden and Wright, 1990; Wright, 1997; Wagener and Brand, 2005; Ben-David et al., 2012). However, there is also evidence that acoustic similarity and temporal coherence may interact. Stream segregation is not achieved instantly, and the time it takes for it to develop depends both on the stimuli used as well as on the listener (Bregman and Campbell, 1971; Bregman, 1990). The segregation of target speech from competing speech appears to take longer than the segregation of target speech from competing noise. Ezzatian et al. (2012) found that segregation of a speech target takes longer to complete when masked by other, competing two-talker speech than when masked by steady-state noise. When younger adult participants were asked to identify a target word presented in a semantically anomalous sentence (aka, for which sentential context could not provide a valid clue to the target word’s identity), there was a relationship between the serial position of the target word in the sentence and recognition accuracy—but only when the masker was competing speech, not when the masker was noise.

### Ageing, Streaming, and Word in Noise Identification

**Figure 1:**
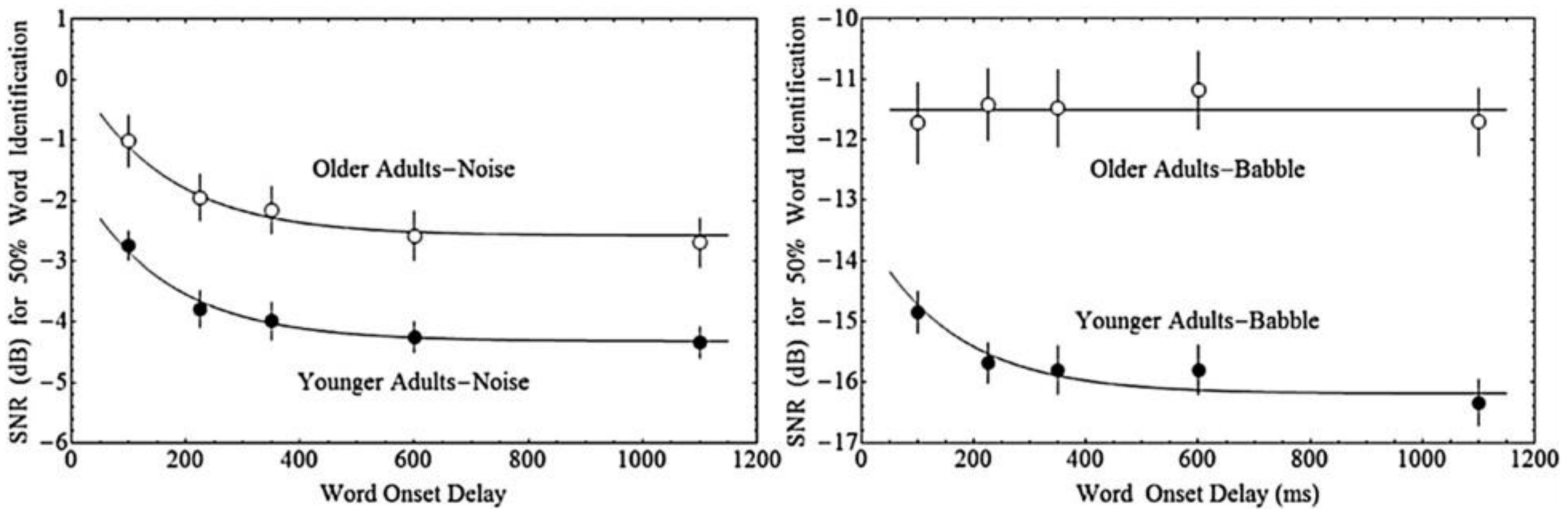
Fifty percent word identification thresholds as a function of the onset delay between the masker and the target word for younger and older adults in either Steady-State Noise, or multi-talker Babble. Adapted with permission from Ben-David et al. (2012).

The impact of both acoustic similarity and temporal factors on auditory stream segregation may change with ageing. Ben-David et al. (2012) asked younger and older adults to repeat single words that were presented with either 100, 225, 350, 600, or 1,100 ms delay from the onset of a masking sound that consisted of either multi-talker babble or steady-state speech spectrum noise. In general, older adults needed a higher signal-to-noise ratio (SNR) to reach 50% word identification accuracy in both maskers, and, in general, longer delays between the target and masker onset resulted in better performance—thresholds decreased exponentially with increased delay between the target and masker. Younger adults, further, exhibited this same relationship between onset delay and performance regardless of masker type (steady-state noise or babble). In contrast, for older adults, target-masker onset delays were only beneficial for the noise masker, where the effect of onset delay was similar for older and younger adults. With babble maskers, older adults appeared unable to take advantage of the delay in onset between the target and the babble (see Figure 1). Hence, while older and younger adults are both able to benefit from onset delay when the masker is noise, only younger adults are able to do so when the masker is babble.

In the present study, we use cortical auditory evoked potentials (CAEPs) to examine if these differences in the ability to use temporal cues to stream segregation are reflected in the very early stages of sensory processing. We focus here on the acoustic change complex (ACC), which is elicited by changes within a continuous stream of sounds. The ACC comprises N1 and P2 deflections, analogous to those elicited by sound onset, and is thought to represent early stages of sensory encoding of the stimulus (e.g., Ostroff et al., 1998; Niemczak and Vander Werff, 2019). If there are age-related differences in the unfolding processes of stream segregation, then we might expect these differences to be reflected in a cortical marker (i.e., the ACC) reflecting sensory registration of the speech stimulus embedded in noise. Prior work suggests that the ACC is responsive to masking of the target; in general, adding a competing auditory source to a speech signal delays the N1 peak onset, and reduces N1 peak amplitude (Billings et al., 2009, 2011). However, there is evidence that attentional factors also affect the N1 response. For example, the amplitude of the N1 wave is often larger when attention is directed to speech sounds than during passive listening (Alain et al., 2004; Billings et al., 2011; Zendel et al., 2015). Modulating a masking noise (vs. a steady-state masker) can result in a CAEP-related release from masking, allowing for a detectable CAEP response for modulated maskers where none exists with a steady-state masker when sounds are presented close to threshold (with a target tone; Androulidakis and Jones, 2006). Introducing interruptions to a masker (vs. a continuous masker) can also affect the CAEP for a speech target relative to a continuous masker (Faucette and Stuart, 2017, 2020). Indeed, there is evidence that the release from masking due to certain characteristics of the stimulus is comparable in magnitude between behavioural and electrophysiological domains. Tanner et al. (2019) examined the CAEPs evoked by a /ba/presented concurrently with either steady-state maskers of 30 and 60 SPL, or a masker that was modulating between the two levels, and examined the electrophysiological and behavioural threshold for detection under all three maskers. The authors found a release from masking of about 13.5 dB in magnitude in both the behavioural and electrophysiological domains. Hence, there is evidence that release from masking can be reflected in changes to the N1 response, and even that these changes may be comparable in magnitude to behavioural release from masking under certain conditions.

The present study used CAEPs to clarify why older adults do not experience an improvement in word recognition with an increase in onset delay between a speech masker and target speech whereas younger adults do. In designing the experiment, we opted to adjust the ratio of the speech target to the masker to produce equivalent word identification scores in all four combinations of Masker Type (Noise vs. Babble) and Onset Delay (100 vs. 600 ms) in both younger and older adults. There were several reasons for doing so. The first reason was to ensure that we would observe a measurable cortical evoked potential. Previous work has suggested it is difficult to measure stable cortical evoked potentials when speech stimuli are presented at threshold levels (in masking: Whiting et al., 1998; Androulidakis and Jones, 2006).

A second reason is that a number of studies have shown that younger and older adults, when tested under identical stimulus conditions, tend to engage different neural mechanisms when performing the same task (see reviews by Wong et al., 2009; Velia Cardin, 2016). However, when there are age-related differences in behavioural performance, it becomes difficult to determine the reasons for any associated age differences in neural activity. It could be, for example, that older adults may need to engage different and/or additional neural mechanisms because hearing and/or visual losses make the task more difficult for them. Or, it could be that there are systemic age-related changes in neural functioning that require that different brain mechanisms and/or areas to be engaged to accomplish the task in older adults irrespective of the level of task difficulty. Adjusting, for example, the SNR to produce equivalent levels of behavioural performance (i.e., equivalent task difficulty), can allow us to distinguish between these two different possibilities. In addition, Alain et al. (2004) found that age-related differences in CAEPs were minimized when participants were attending to the auditory stimulus, and performing at equivalent behavioural levels. Hence, the disappearance of age-related differences in neural activity when age-related differences in behavioural performance are eliminated (for instance, by adjusting the SNR) would be consistent with the notion that younger and older adults engage the same neural mechanisms when task difficulty is adjusted to produce equivalent behavioural performance. On the other hand, a finding that age-related neural processing differences persisted after equating younger and older adults with respect to behavioural output, would be consistent with the notion that older adults must engage different neural processes to perform a task. The latter result would suggest that the neural circuitry available to younger adults when required to perform a certain task, such as unmasking an auditory target, is not as available to older adults as it is to younger adults.

A similar argument could be made with respect to the engagement of different brain mechanisms when there is a change in task (e.g., a change in the Onset Delay between masker and speech target). If, after adjusting for behavioural performance across the two delays, we find differences in CAEPs, we can conclude that there are neural processing differences between the two delay conditions that are relatively independent of behavioural performance. Finally, we might find an interaction between masker similarity, onset delay, and age that will be easier to interpret if behavioural performance is equated across all eight combinations of these three factors.

Hence, in the present study, we were searching for neural evidence of the behavioural result that an increase in Onset Delay between a babble masker and a speech target makes listening easier for younger adults but not for older adults. We conducted this search when both age groups had equivalent word identification scores. Any differences in early cortical responses under such circumstances would indicate an age difference in the way speech in babble was processed.

## Materials and Methods

### Participants

**Figure 2:**
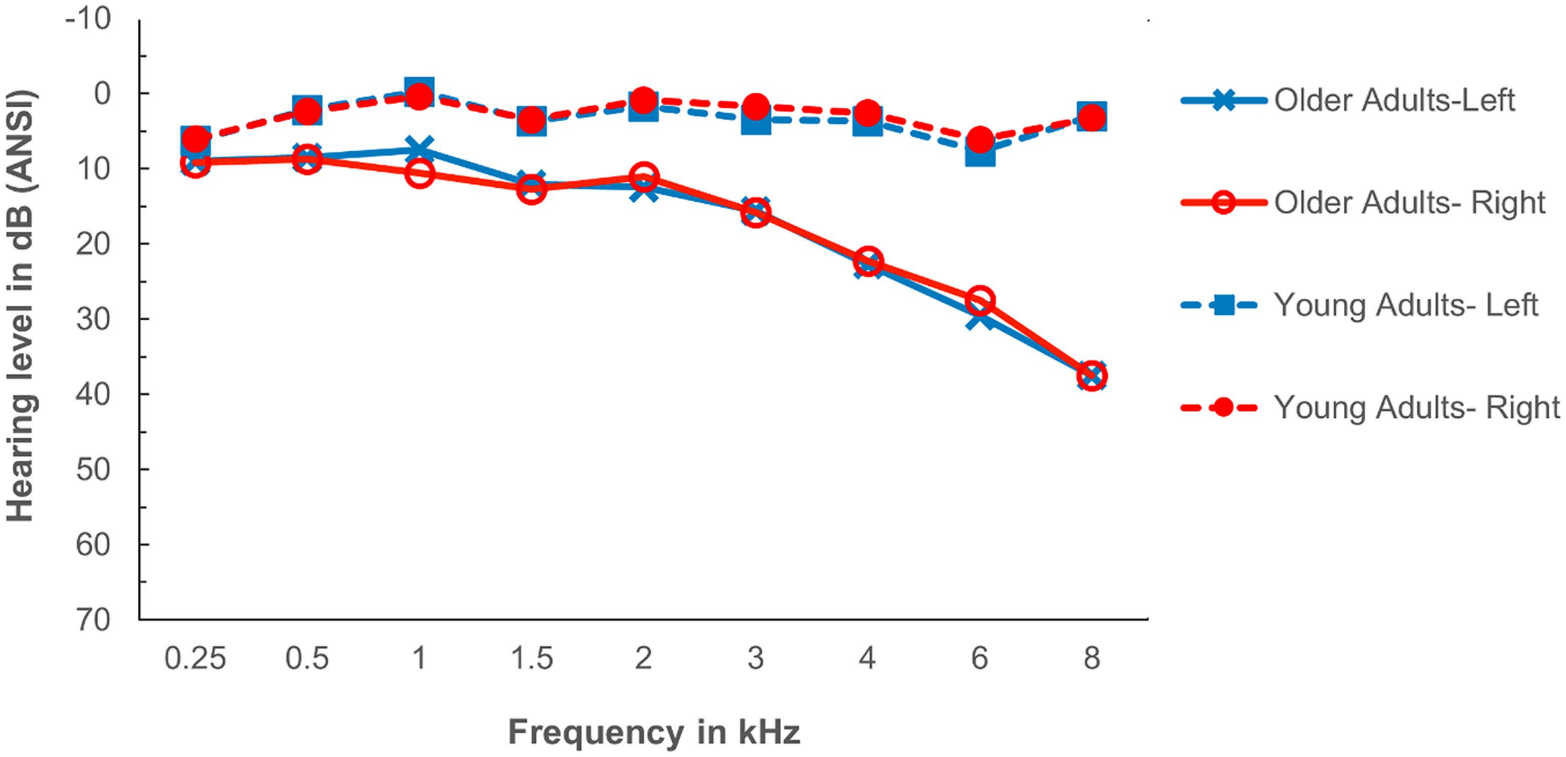
Average audiograms for the two age groups (Young vs. Older adults). Left and right ears are plotted separately.

Twenty-four younger adults (*M*_age_ = 21.6; *SD*_age_ = 2.7; range = 18–27 years) and 24 older adults (*M*_age_ = 72.5; *SD*_age_ = 5.7; range = 65–85 years) received a modest stipend in exchange for participation in this study. Younger adult participants were students at the University of Toronto Mississauga; older adults were community-dwelling volunteers. All participants indicated they were native speakers of English who were not fluent in any additional languages, and achieved a minimum score of 9/20 on the Mill Hill vocabulary test (Raven, 1965). Hearing screenings conducted within the year prior to participation showed that all participants had pure-tone air-conduction thresholds within clinically normal limits between 200 and 3,000 Hz (see average hearing thresholds presented in Figure 2). In addition, all participants indicated *via* self-report that they were in good health with no history of auditory pathology or neurological trauma. We were unable to obtain readable CAEPs in one younger adult and this participant was excluded from the ERP analysis.

### Stimuli and Apparatus

Five hundred and sixteen bi-syllabic recorded words, spoken by a female actor with a southern Ontario accent, taken from Murphy et al. (2000), were used for this experiment. Words were divided into four lists of 128. The word lists were derived from 10 lists featuring the same words, as used in Ben-David et al. (2012) in that the first eight word lists from this study were combined, and the remaining two lists were split in half and added to each list. Thus, the lists were well equated on word frequency, density of lexical neighbourhood, and duration (for further details, see Ben-David et al., 2012). Spoken words were presented to participants on a background of either continuous speech spectrum noise or multi-talker babble taken from the “Revised Speech Perception in Noise” (R-SPIN) test (Bilger et al., 1984). All of the 516 words were equated with respect to root mean square amplitude. Word stimuli were delivered binaurally by converting the digital signal to analogue form (using a 16-bit digital-to-analogue converter TDT DD1), and controlling the analogue output using an Enhanced Real-time Processor (TDT RP2.1) and programmable attenuator (TDT PA5), before delivering the signal to a headphone buffer (TDT HB7) and a Sennheiser HD 265 headphone.

### Procedure

Participants were tested individually in a single-walled sound attenuated booth. Each experimental session consisted of four blocks; each block consisted of 128 consecutive single-word trials. For two of the blocks, participants heard the target words masked by multi-talker babble; in the other two blocks, participants heard target words masked by speech-spectrum noise. For each masker type (babble and noise), participants completed one block with a 100 ms delay between the onset of the masker and the subsequent onset of the to-be-repeated word, and one block with a 600 ms delay between the onset of the masker and the subsequent onset of the to-be-repeated word. The order of presentation of the four possible masker/delay conditions (Babble 100 ms; Babble 600 ms; Noise 100 ms; Noise 600 ms) was counterbalanced across participants, with each participant completing a randomly assigned block order set, such that each of the four possible conditions was presented at each of the four possible serial block locations (1st, 2nd, 3rd, or 4th) an equal number of times within each age group, and across the entire sample. The four word lists were always presented in the same order across all participants. In this way, each individual word list was presented in each of the four possible masker/delay combinations an equal number of times.

Participants were told to repeat the word they heard and encouraged to guess if they were somewhat uncertain, but say “pass” if they were very unsure of the word. Participants were not given practice trials or feedback. Optional short breaks were permitted between each of the four blocks. Accuracy was coded during the experimental session by a native English-speaking experimenter who listened to participant responses *via* headphones. After recording the participant response, the experimenter then cued the next word, with a minimum of 250 ms between the end of the participants’ utterance and the beginning of the next stimulus in the set.

Words were always presented at 60 dB SPL. The levels of the competing speech or noise were determined according to the following procedure. The psychometric functions reported in Ben-David et al. (2012) were used to determine the SNR that produced a level of 95% correct word recognition for the two groups of participants (young and old) at each of the four conditions in this experiment. These SNRs reflect the average level at which participants in each age group, at each masker-target onset delay, were 95% accurate at determining the target word in each condition, rounded to two decimal places. For younger adults, these levels, in dB, were: Noise_100 ms_: +6.93; Noise_600 ms_: +4.95; Babble_100 ms_: −1.65; Babble_600 ms_: −2.70. For older adults, these levels, in dB, were: Noise_100 ms_: +9.41; Noise_600 ms_: +7.03; Babble_100 ms_: +3.41; Babble_600 ms_: +1.46.

### Electrophysiology Recording

Neuroelectric brain activity was recorded continuously using a 128-channel HydroCel Geodesic Sensor Net (EGI technology) with a sample rate of 500 Hz. During recording, data were referenced to Cz with a bandpass of DC-100 Hz, and stored for offline analysis. EEG recordings were preprocessed offline using Brain Electrical Source Analysis software (BESA Research version 7.0; MEGIS GmbH, Gräfelfing, Germany).

### EEG Preprocessing

All trials, regardless of behavioural accuracy, were included in the EEG analysis. The EEG data were visually inspected to identify segments contaminated by defective electrodes. Noisy electrodes were interpolated using data from the surrounding electrodes, and no more than eight electrodes were interpolated per participant. The EEG was then re-referenced to the average of all electrodes and digitally filtered with a 1 Hz high-pass filter (forward, 6 dB/octave) and 30 Hz low-pass filter (zero phase, 24 dB/octave), which was identical to the filters used by others (Billings et al., 2011). For each participant, a set of ocular movements was identified from the continuous EEG recording and used to generate spatial components to best account for eye movement artefacts. The spatial topographies were then subtracted from the continuous EEG to correct for lateral and vertical eye movements as well as for eye blinks. The data were parsed into 700 ms epochs that were time-locked to either noise onset or word onset, including 100 ms of pre-stimulus activity (which served as the baseline). Epochs with EEG signal exceeding ±60 μV were marked and excluded from further analysis. The processed data consisted of a minimum of 75% of the epochs per experimental condition and participant for the young and older adult group. The epochs were averaged according to the experimental conditions: babble noise, speech-spectrum noise; 100 ms noise preceding word onset; and 600 ms noise preceding word onset. Each average was then baseline-corrected with respect to the 100 ms pre-stimulus baseline interval.

The effect of noise type and noise duration on CAEP amplitude and latency was quantified using 15 electrodes over the midline central and fronto-central scalp area. This cluster of electrodes best capture the dominant (i.e., tangential orientation) source activity for N1 and P2 waves from the auditory cortices located in the superior temporal gyrus. For the 600 ms noise duration, the N1 and P2 was measured related to word onset. The N1 peak latency and amplitude was defined as the maximum negativity between 100 and 250 ms. The P2 peak latency and amplitude was defined as the maximum positivity between 200 and 400 ms.

For the 100 ms delay between masker and word onset, the N1 and P2 waves elicited by noise onset partly overlapped with those elicited by the word because of the short delay between masker and word onset. To isolate the response to the word as much as possible, we subtracted the auditory evoked responses elicited in the 600 ms condition from the 100 ms condition. The masker onset and masker duration were identical in both conditions, with the only difference being the presence of a spoken word starting at 100 ms in the 100 ms condition. This subtraction procedure is based on the assumption that the auditory evoked responses elicited by the noise and word onset sum together linearly. The difference wave is thought to index processing of the masked word in the 100 ms condition, with the response related to masker onset removed. We then measured the N1 and P2 peak latency and amplitude from this difference waveform. The N1 peak latency and amplitude were defined as the maximum negativity between 200 and 350 ms after masker onset. The P2 peak latency and amplitude were defined as the maximum positivity between 300 and 500 ms after masker onset. Because the word was presented 100 ms after masker onset, we subtracted 100 ms from the peaks found in the difference waveform to allow comparison with the 600 ms delay condition.

## Results

### Behavioural Results

After each experimental session, a native English speaker scored the accuracy of each participant by listening to an audio recording of each session. Average percentage agreement for the online coding by the experimenter and the offline coding by the second scorer was 98.5% for younger adults (min = 93.8%) and 98.7% for older adults (min = 93.0%). For individual word trials where the two accuracy scores disagreed, a third rater listened to the recording and the judgement (correct or error) endorsed by two out of the three scorers was used; these resolved accuracy totals were retained as the accuracy scores for each individual. Average percentage correct for each age group and condition are displayed in Table 1.

**Table 1:** Average percentage correct word identification performance by Condition (Noise or Babble) and Delay (100 or 600 ms) for all 24 Older and 24 Younger Participants.

	Noise		Babble	
	100 ms	600 ms	100 ms	600 ms
Younger Adults	96.97	95.80	96.84	96.45
Older Adults	95.55	94.53	95.61	94.76

Participants from both age groups performed within two percentage points of 95% correct for all conditions. Since word identification performance for all groups was centred at an extreme end (i.e., 95% correct) of the percentage scale, a Stevens arcsine transform was used to convert word identification performance into sau units (see Sherbecoe and Studebaker, 2004). A 2 (masker type) by (2 onset delay) by 2 (age group) ANOVA was performed on these transformed values with Onset Delay and Masker Type as within-subject factors, and Age Group as a between-subjects factor. This analysis revealed no main effect of masker, *F*(1,46) = 1.041, *p* = 0.313, indicating that word identification score did not differ overall across the two types of maskers. There was a main effect of delay, *F*(1, 46) = 16.64, *p* < 0.001, reflecting that word identification performance—collapsing across masker type and age group—was statistically significantly higher in the 100 ms delay condition (*M* = 87.29, *SE* = 0.478) than the 600 ms delay condition (*M* = 85.93, *SE* = 0.448); however, the magnitude of this discrepancy equates to a difference in word identification accuracy of less than one word out of the 128 total words per list. This effect of delay, additionally, did not vary between the age groups, *F(1,46) <* 1, nor between masker types, *F(1,46) =* 2.089, *p* = 0.155. There was a significant main effect of age group on overall word identification performance, *F(1,46)* = 4.48, *p* = 0.04, reflecting that older adults word identification performance (*M* = 85.69, *SE* = 0.611), collapsing across maskers and delays, was slightly worse than younger adults (*M* = 87.53, *SE* = 0.611). Again, however, the magnitude of this difference was such that older adults, collapsing across masker condition and delay duration, identified on average only 1.79 fewer words per condition block than younger adults. The effect of age group on word identification performance did not differ across the masker types, *F(1, 46)* < 1, *p* = 0.907, or onset delay (see above). The three-way interaction between masker, delay, and age group was also not significant, *F(1,46) <* 1.

### Electrophysiological Results

The impetus for this study was to search for electrophysiological correlates of the effects of release from masking due to an increase in the onset delay between masker and target words (from 100 to 600 ms) on the initial processing of words heard in two kinds of acoustic interference (noise vs. babble) for younger and older adults. A previous behavioural study found a significant release from masking with an increase in onset delay for young adults in Noise, young adults in Babble, and older adults in Noise, but not for older adults in Babble (Ben-David et al., 2012).

**Figure 3:**
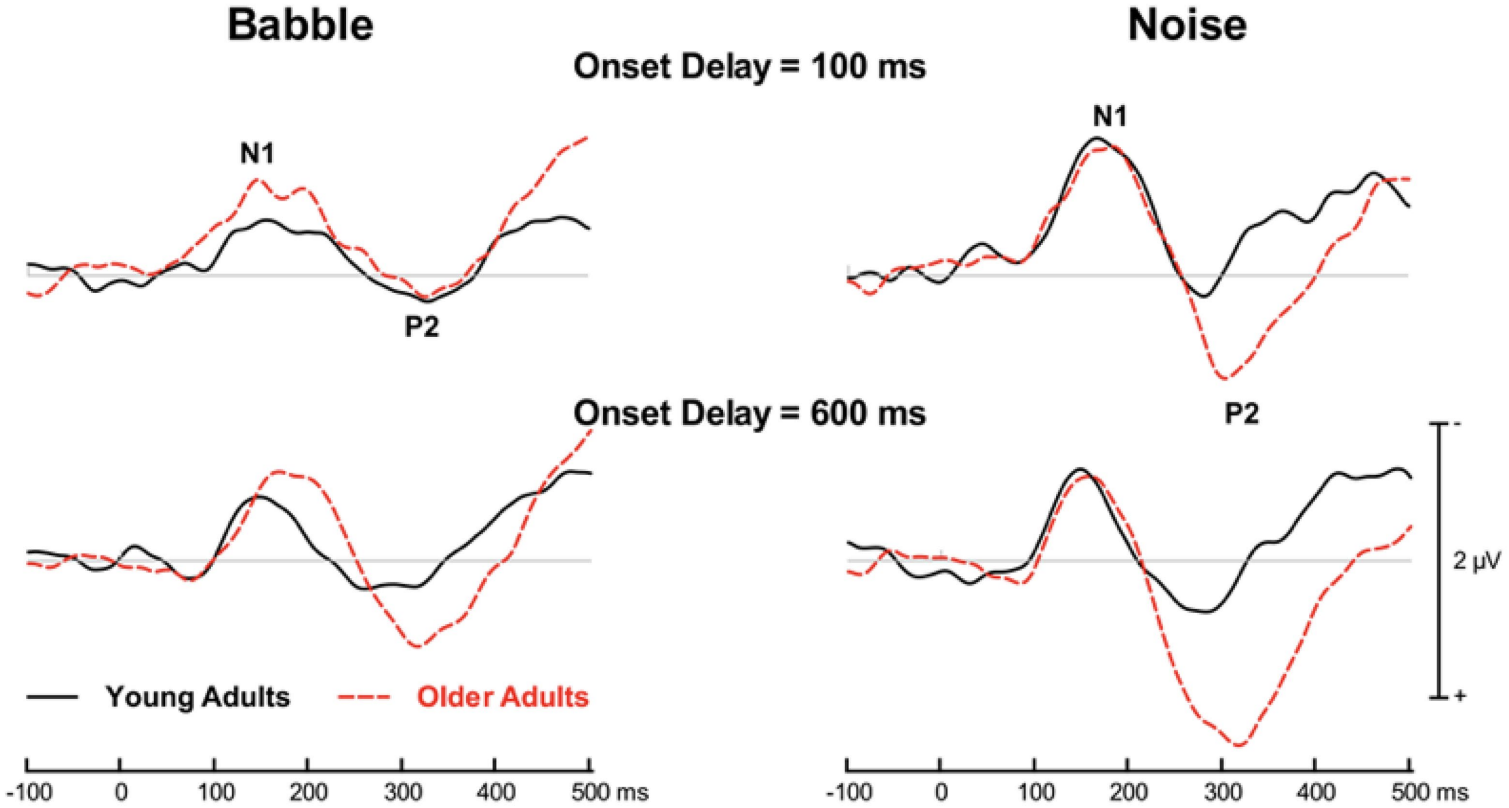
Group mean event-related potentials time-locked on word onset. Traces are shown when the target word was preceded by 100 ms of masker (top row) or 600 ms of masker (bottom row). The electrophysiological responses to the target word embedded in babble are shown on the left whereas those obtained when the word was embedded in speech spectrum noise (i.e., noise) are shown on the right.

In both groups and in all experimental conditions, words presented with a masker generated an ACC that comprised N1 and P2 waves that peaked at central sites. Figure 3 shows the group mean ACC from the midline fronto-central electrode in young and older adults as a function of the experimental conditions.

When the word is masked by Noise at either Onset Delay, there does not appear to be any age differences with respect to the N1 peak. With respect to the P2 wave, it appears to peak later in older than in younger adults with the amplitude of the peak being greater in older adults at both Onset Delays. When the masker is Babble and the Onset Delay is 100 ms, the N1 peak latency does not appear to differ between younger and older adults, although the magnitude of the N1 peak appears to be larger in older adults. There also does not appear to be any significant differences in the location and amplitude of the P2 in Babble when the delay is 100 ms. However, when the Onset Delay is 600 ms, both the N1 and P2 waveforms appear to peak later in older than in younger adults with slightly higher amplitudes in both cases.

The traces in Figure 3 indicate that both the amplitude and locations of the N1 and P2 peaks differ with respect to the Age Group to which participants belong, and that the extent of this difference differs with both the Type of Masker and Onset Delay. Hence, the degree of release from masking that presumably occurs with an increase in Onset Delay from 100 to 600 ms in both maskers for young adults, but only in Noise for older adults, can differentially affect both the time between the onset of the word and the peak of each wave (its latency), as well as the amplitude of the electrophysiological response at its peak (its amplitude). Estimates of these two parameters of N1 and P2 were obtained as described in the Methods Section. Because the factors in this experiment can affect the two parameters of the waveforms in different ways, we looked for a way to simultaneously represent both parameters of a waveform together.

**Figure 4:**
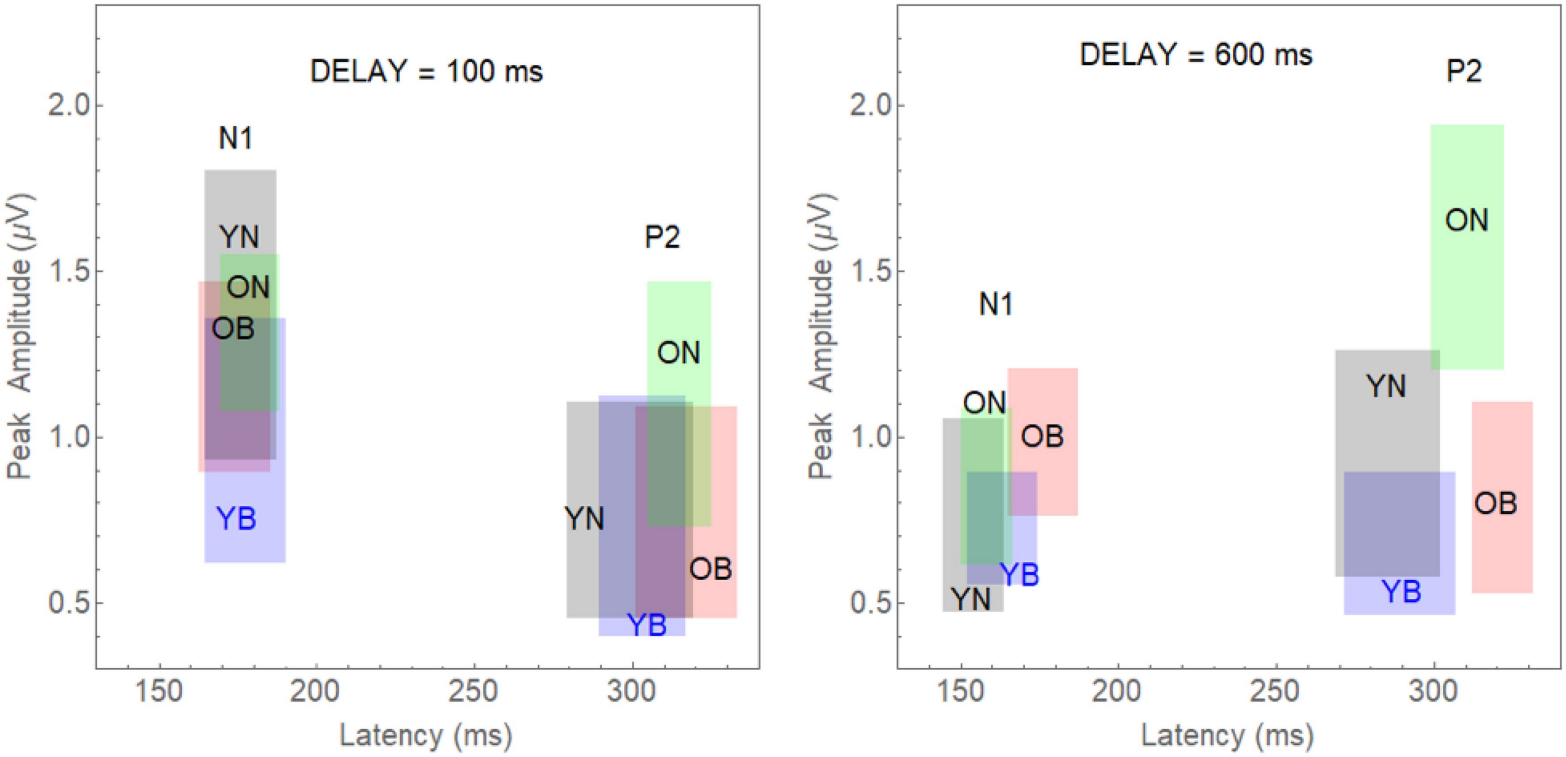
The 95% joint CI rectangles for latency and amplitude of the N1 and P2 waves in Younger and Older Adults for the Noise and Babble Maskers and 100 and 600 ms Delays. **Left-panel**: 95% CI rectangles for a target word onset delay of 100 ms. Separate rectangles are shown for the two Age Groups reporting heard words in both Noise and Babble for the two different waves (N1 and P2). The grey rectangles depict the CIs for young adults in noise (YN); the blue rectangles are for young adults in babble (YB). The green rectangles are for old adults in noise (ON), and the red rectangles are for old adults in babble (OB). **Right-panel**: The equivalent rectangles for a target word onset delay of 600 ms.

Specifically, we computed 95% confidence limits for both N1 and P2 latency and amplitude in each group by experimental condition combination: (young-noise-100, young-noise-600, young-babble-100, young-babble-600, old-noise-100, old-noise-600, old-babble-100, and old-babble-600). For example, to represent the joint effects of latency and amplitude on N1 for young adults in Noise, at a delay = 100 ms, we constructed a rectangle in a two-dimensional plot whose *x*-axis was the latency of the peak of the N1 wave relative to the onset of the target word, and whose *y*-axis was its amplitude. This rectangle is labelled as YN and appears in grey in the left-hand panel of Figure 4 in the section reserved for the N1 waveform. The 95% CI for latency is specified by the *x* coordinate of the right-hand side of the grey rectangle minus the *x* coordinate of the left-hand side of the rectangle. The 95% CI for peak amplitude is specified by the difference between the *y* coordinate of the upper boundary of the grey rectangle minus its lower boundary. The probability that both the population mean latency and population mean peak for this group fall within this rectangle is 0.95*0.95 = 0.9025.

Now if two of the groups had the same population mean latency and the same population mean peak amplitude, we would expect considerable overlap between the two rectangles. Conversely, if the two groups had significantly different population mean latencies, and/or significantly different population mean peak amplitudes, we would expect to find very little overlap between the rectangles for these two groups.

In the rest of the left panel of Figure 4, we plot the eight rectangles representing the joint CIs for latency and amplitude when the target word was presented with a Delay = 100 ms for combinations of 2 Age Groups (Young-Old) × 2 Maskers (Noise, Babble) × 2 waveforms (N1, P2). The right-hand panel of Figure 2 plots the rectangles for the same eight combinations when the delay was 600 ms. The left-panel shows that there is considerable overlap at an onset delay of 100 ms among the four groups for both the N1 and P2 waves.

Given that we have adjusted the SNR to produce equivalent behavioural results in both groups and masker types for the N1 and P2 waves, this is what we would expect if the amount of release from masking (if any) at a delay of 100 ms were the same in all cases. The results for a word onset delay of 600 ms differ substantially from those found for an onset delay of 100 ms. First, for the N1 wave, the CI rectangles for three of the rectangles (young and older adults in noise, and younger adults in babble) appear to overlap substantially among each other, with all three of them overlapping with the rectangle representing older adults in babble to a much lesser extent. Second, for the P2 wave, the CI rectangles for young adults in both babble and noise overlap with one another, with neither overlapping with either older adults in noise or in babble. In addition, the latter two rectangles (older adults in noise or in babble) also do not overlap with one another. Table 2 quantifies the amount of overlap among the four rectangles comparing N1 and P2 outcome measures for both delays of 100 and 600 ms (see Appendix for how this was computed). For example, for N1, at an Onset Delay of 100 ms, the first entry in the cell defined by row YN and column YB (0.432) specifies the probability of finding both of the population means for the YN group within the CI rectangle corresponding to YB group ( $p\left[\mu_{x,YN}\mu_{y,YN}\right]$ falling in the overlap of the confidence rectangle for the YN with the confidence interval for YB). The second entry in that cell (0.543) specifies the probability of finding both of the population means for the YB group within the confidence interval rectangle corresponding to YN group ( $p\left[\mu_{x,YB}\mu_{y,YB}\right]$ falling in the overlap of the confidence rectangle for the YN with the confidence interval for YB). In general, the top entry in each of the cells in the table specifies the probability of the joint appearance of the population means for the row condition appearing in the confidence rectangle for the column condition ( $p\left[\mu_{x,rowcondition}\mu_{y,rowcondition}\right]$ falling in the overlap of the confidence rectangle for the row condition with the confidence interval for the column condition). The bottom entry specifies the probability of the joint appearance of the population means for the column condition appearing in the CI for the row condition ( $p\left[\mu_{x,columncondition}\mu_{y,columncondition}\right]$ falling in the overlap of the confidence rectangle for the row condition with the CI for the column condition). These two probabilities can range between 0 and 0.9025 (0 if there is no overlap between YN and YB, and 0.9025 if the CI rectangles are identical for YN and YB). The actual degree of overlap shown in Figure 4 for these two rectangles is based on the confidence intervals constructed from the results of 23 younger adults tested in both Babble and Noise. As such this confidence interval rectangle will vary from experiment to experiment. In the Appendix, we derive the probability values expected under the null hypothesis when the population means for YN and YB are identical. In order to reject this null hypothesis at the α=0.05 level requires that these two probabilities are less than 0.059. Clearly, this is far from being the case in the present example.

**Table 2:** The degree of overlap of the Pairs of CI Rectangles at two delays for two different waveforms.

N1: Delay = 100				P2: Delay = 100			
	YB	ON	OB		YB	ON	OB
YN	0.432 0.543	0.601 0.888	0.607 0.863	YN	0.752 0.877	0.152 0.386	0.382 0.552
YB		0.237 0.588	0.586 0.805	YB		0.206 0.334	0.563 0.440
ON			0.782 0.563	ON			0.435 0.453
N1: Delay = 600				P2: Delay = 600			
	YB	ON	OB		YB	ON	OB
YN	0.469 0.486	0.642 0.820	0.000^***^ 0.000^***^	YN	0.377 0.718	0.001^***^ 0.001^***^	0.000^***^ 0.000^***^
YB		0.618 0.557	0.095^*^ 0.057^**^	YB		0.000^***^ 0.000^***^	0.000^***^ 0.000^***^
ON			0.016^**^ 0.012^**^	ON			0.000^**^ 0.000^**^

*YN is Young-Noise, YB is Young-Babble, ON is Old-Noise, and OB is Old-Babble*.

* *α* *= 0.10;*

** *α = 0.05;*

*** *α = 0.025*.

An examination of Table 2 confirms the visual impression that when the onset delay is 100 ms, we cannot reject the null hypothesis that the intersection of the confidence interval rectangles for any of the pairwise comparisons among the four rectangles occurs because the two groups have the same population mean latency and population mean peak amplitude. This holds for both N1 and P2.

**Figure 5:**
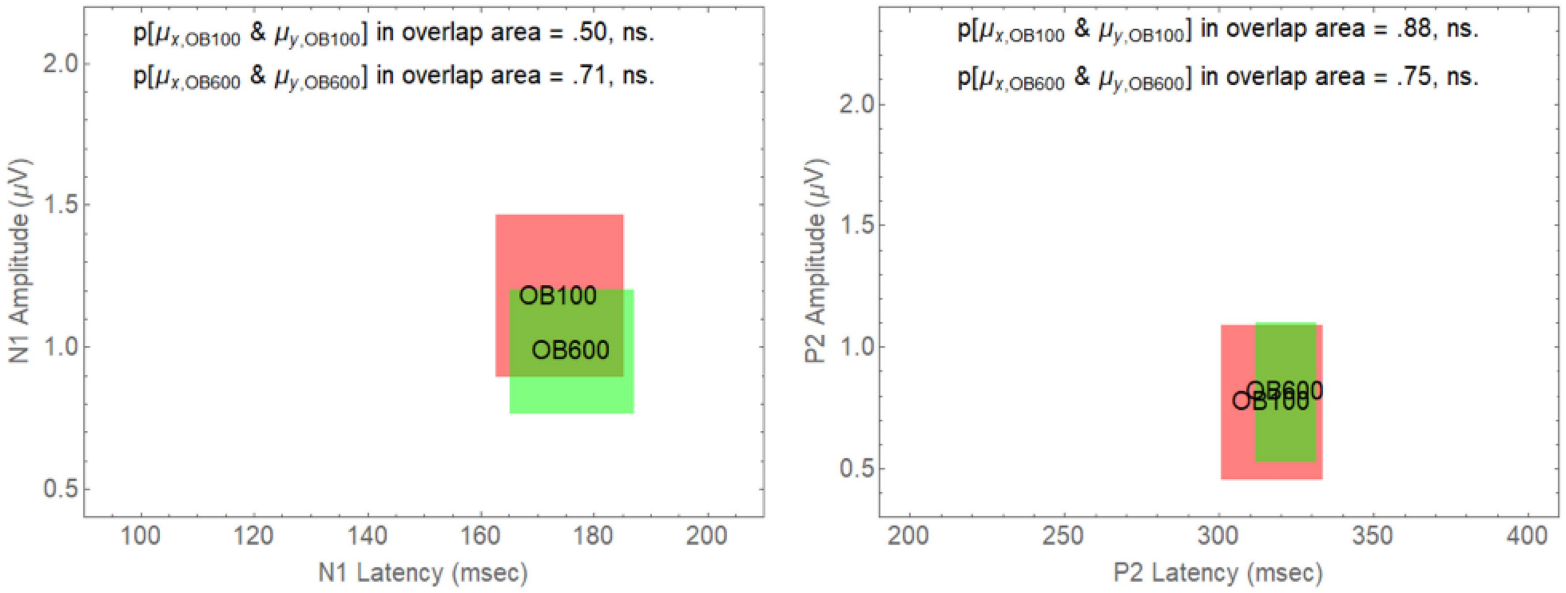
Overlap between the CI rectangles for the 100 and 600 ms delay conditions for older adults in Babble. **Left Panel**: CI rectangles for older adults in the Babble condition for onset delays of 100 and 600 ms for the N1 wave. **Right Panel**: CI rectangles for older adults in the Babble condition for onset delays of 100 and 600 ms for the P2 wave.

However, for an onset delay of 600 ms, the null hypothesis is rejected in all of the comparisons involving older adults in babble for the N1 wave. For the P2 wave, the null hypothesis is rejected for all of the comparisons except for the comparison of the YN and YB CI rectangles. To identify the reasons for this result, we note that in Figure 4, going from on onset delay of 100–600 ms appears to shift three of the rectangles (YN, YB, and ON) away from the position occupied by the CI rectangle for older adults in Babble, which appears to maintain its position for both the N1 and P2 waves. To confirm this visual impression from Figure 4, we plotted, in Figure 5, the locations for the rectangles for older adults in babble for onset delays of 100 and 600 ms for N1 (left panel) and P2 (right panel). This figure indicates that we cannot reject the null hypothesis that the CI rectangle for older adults in babble is independent of onset delay for both for the N1 and P2 waves.

**Figure 6:**
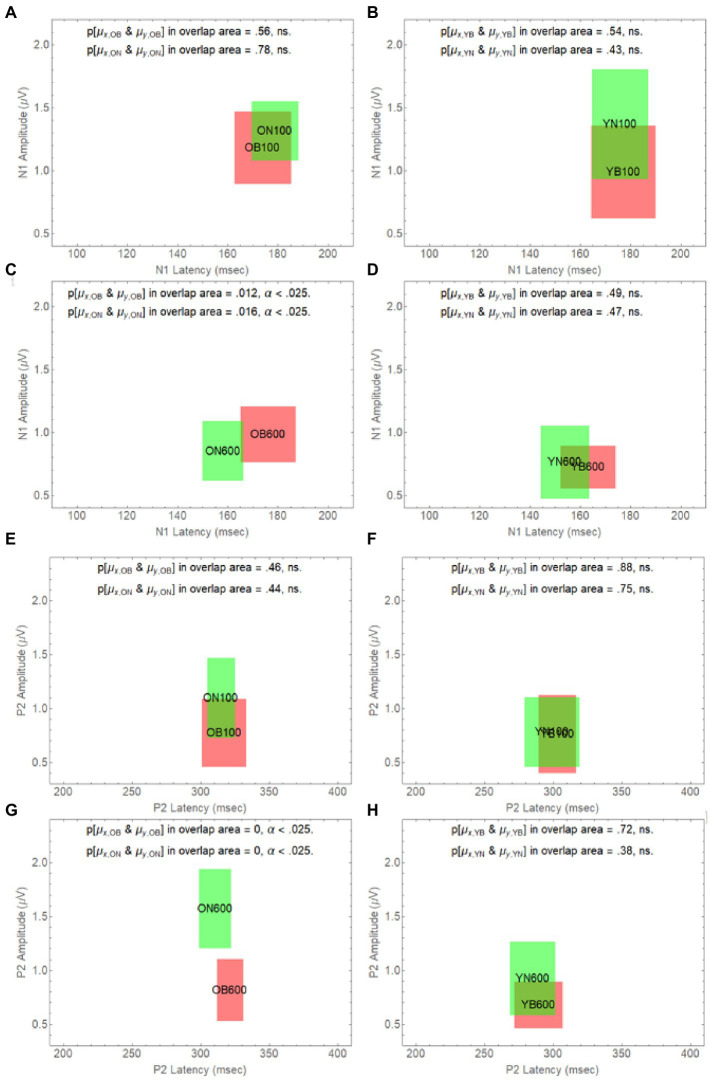
Degree of overlap between Noise and Babble for Onset Delays of 100 and 600 ms for N1 and P2 waves in Younger and Older Adults. The overlap between masker condition (N vs. B) is depicted in separate panels **(A-H)** for all possible combinations of delay (100 vs. 600) and age (O vs. Y), for the two waveform components (N1 and P2).

To further explore how the difference in onset delay differentially affects younger and older adults, in Figure 6, we have plotted how age and onset delay affect the confidence rectangles of older adults for the N1 wave (panels A–D) and the P2 wave (panels E–H). Panel A and B shows that for both younger and older adults we cannot reject the null hypothesis that the population means in Noise are the same as they are in Babble when the onset delay is 100 ms. We also cannot reject this null hypothesis for younger adults for an onset delay of 600 ms (panel D). However, the null hypothesis is rejected for older adults at a delay of 600 ms (panel C). Panels E–H show that this same pattern holds for younger and older when the P2 wave is considered.

Figures 5, 6 considered together are consistent with the following hypothesis derived from the behavioural results relating thresholds to delay in younger and older adults. If increases in onset delay do not release older adults from masking when the masker is Babble, then early cortical processing (the N1 and P2 waves) of target words in Babble for older adults should be unaffected by delay as is found in Figure 5. In addition, if increases in onset delay releases older adults in Noise but not in Babble, then the CI rectangle should be different for older adults in Noise than it is for them in Babble when the onset delay is 600 ms. However, the CI rectangles for older adults in noise should be equivalent to those in Babble when the delay is 100 ms because there is no release from masking for either Noise or Babble for a delay of that magnitude. Moreover, this prediction should hold true for both N1 and P2 waves. Conversely, because an increase in onset delay releases younger adults from masking for both Noise and Babble maskers, we would not expect CI rectangles to differ significantly for Noise and Babble maskers as a function of delay and ACC components (i.e., N1 and P2) in younger adults. Figure 6 confirms this prediction.

## Discussion

### Prediction Derived From a Release From a Masking Model for Early Cortical Processing of the Acoustic Signal

We have derived several predictions concerning the results of the present experiment based on the data from Ben-David et al. (2012). Those investigators measured 50% thresholds for the identification of words in two kinds of maskers (Babble and Steady-State Noise) as a function of the delay between the onset of the masker and the onset of the target word for both younger and older adults (Figure 1). Older adults require greater SNR at all Onset Delays tested for both types of maskers (see the section “Materials and Methods”). Thresholds decrease with increasing Onset Delay for young adults in both kinds of maskers, but only in Noise maskers for older adults. For older adults in a Babble masker, thresholds do not change as a function of Onset Delay.

Recall that, in the present experiment, SNR were adjusted to produce a word identification rate of approximately 95% in all four conditions in both younger and older adults. Now suppose that in the Onset Delay = 100 ms conditions, this SNR manipulation resulted in word identification being equally difficult in the babble and noise maskers for both younger and older adults for both waveforms as suggested by the Ben-David et al. (2012) results. In that instance, we would expect the early cortical processing of the acoustic signal (the N1 and P2 waves) to be identical. However, given the Ben-David et al. (2012) results, we would expect that the word-identification for younger adults should be easier for an Onset Delay of 600 ms than for an Onset Delay of 100 ms for both Noise and Babble Maskers, and for older adults only when the masker is Noise. Hence, word-identification difficulty should be equivalent in the following conditions: (1) Young adults in Noise, Onset Delay = 100 ms; (2) Young adults in Babble, Onset Delay = 100 ms; (3) Older adults in Noise, Onset Delay = 100 ms; (4) Older adults in Babble, Onset Delay = 100 ms; and (5) Older adults in Babble, Onset Delay = 600 ms. Moreover, these predictions should hold true for both N1 and P2 waves.

To evaluate these predictions, we constructed 95% CIs for the population means for latency and peak amplitude in each of the eight combinations of Age Group (Young, Old), Onset Delay (100 vs. 600 ms), and Type of Masker (Noise Babble) for both N1 and P2 waves. The two confidence intervals for a Group were used to define CI rectangles for that group in a two-dimensional space where the abscissa (*x*-axis) is the latency of the peak cortical response (either N1 or P2 waves) from word onset, and the ordinate (*y*-axis) is the amplitude associated with the peak in question. In this two-dimensional space, the width and location of the rectangle along the latency dimension corresponded to the CI for latency, and the extent and location of the rectangle along the amplitude dimension corresponded to the CI for peak amplitude.

Figure 4 plotted these CI rectangles in the two-dimensional space where the *x*-axis is the latency of the response (the time from word onset to the peaks of either the N1 and P2 wave), and the *y*-axis is the amplitude of the respective peaks. The degree of overlap among the four rectangles (YN, YB, ON, and OB) in Figure 4 (delay = 100 ms) and the associated analysis supports the prediction that the early cortical responses (N1 and P2) are similar among these four rectangles. Table 2 shows that the null hypothesis that the population means for latency and peak amplitude are the same for each of the six pairings of these rectangles cannot be rejected for either the N1 and P2 waves when Onset Delay = 100 ms. The fact that the CI rectangles for N1 and P2 waves for Older adults in Babble for a 100 ms Delay overlap those for the same rectangles for a 600 ms delay is consistent with the prediction that word identification is equally difficult for Older adults in Babble for a 600 ms Delay as it is for Older Adult in Babble when the Delay is 100 ms (see Figure 5).

Table 2 also indicates that, for the N1 wave at an onset delay of 600 ms, the overlap is significantly diminished when the OB group is compared to the other three groups, but that the null hypothesis cannot be rejected that the same population means for latency and amplitude can account for each pairing of the remaining three rectangles (YN, ON, and YB). Finally, the Ben-David et al. (2012) data predict that word identification difficulty should be the same for younger adults in both Noise and Babble when the Onset Delay = 100 ms because there is no release from masking with this Onset Delay. The Ben-David et al. (2012) results also predict that word identification difficulty should be the same for younger adults in both Noise and Babble when the Onset Delay is 600 ms because a 600 ms Onset Delay releases younger adults from masking for both Noise and Babble maskers. For older adults, the Ben-David et al. (2012) results predict the same for older adults when the Onset Delay is 100 ms because there is no release from masking for this Onset Delay for both types of maskers. However, when the Onset Delay = 600 ms, word identification should be easier for a Noise masker than for a Babble masker because there is a release from masking for older adults when the masker is Noise, but not when the masker is Babble. The CI rectangles in Figure 6 support this prediction. For younger adults, there is significant overlap for Babble and Noise maskers for both Onset Delays. However, for older adults, while there is significant overlap between CI rectangles for Noise and Babble maskers for Onset Delay = 100 ms, the null hypothesis that the same population means can account for both Noise and Babble maskers when the Onset Delay is 600 ms is rejected, indicating that cortical processes for these two maskers is not the same when the Onset Delay is 600 ms. For older adults at Onset Delay 600 ms, the CI rectangle for a noise masker when examining the N1 wave occurs at a shorter latency than that that for the Babble masker. When examining the P2 wave, older adults at Onset Delay = 600 ms have higher amplitudes when the Masker is Noise than when it is Babble. This may reflect difference in attentional allocation or listening effort, with older adults paying more attention when the word is embedded in babble than in noise. This account is consistent with prior research indicating that the P2 amplitude is larger when words are familiar or could easily be identified (e.g., Faucette and Stuart, 2017).

### Potential Cautions Associated With These Analyses

As noted in the section “Materials and Methods,” the analyses related to Onset Delay = 100 ms are based on a difference waveform in that the first 600 ms of the ERP waveform when the Onset Delay was 600 ms was subtracted from the ERP waveform to when the Onset Delay was 100 ms. The failure to find any differences among the four groups in this difference waveform could be attributed to the increased variability in this difference waveform due to the subtraction process. In this experiment, two measures (latency and amplitude) were collected for each of two waveforms (N1 and P2) for two Age Groups attempting to identify words masked by either Noise or Babble when the Onset Delay was set to 100 ms. Hence, there were 16 measures of performance taken at an Onset Delay of 100 ms that were based on a difference waveform. There were also 16 measures of performance taken when the Onset Delay was set to 600 ms. For these 16 measures, we computed the ratio of the variance of each measure taken using an onset delay of 100 ms to its corresponding measure taken using a delay of 600 ms. If measures taken at an Onset Delay of 100 ms are more variable than comparable measures taken at an Onset Delay of 600 ms, their ratios of their variances should be greater than 1.0. Of the 16 measures, the ratio of variances was greater than 1 in 12 of them, which is significant at the 0.05 level (two-tail test). However, the average ratio of the 16 variances was only 1.66, suggesting that although there is increased variance for the measures taken with an Onset Delay of 100 ms when compared to the variance of measures taken with an Onset Delay of 600 ms, the increase in variance is not very large.

Nevertheless, the increased variance of the measures taken with an Onset Delay = 100 ms relative to the comparable measures taken with an Onset Delay = 600 ms could be responsible, in part, for the fact that no differences were found among the four groups in both N1 and P2 waves when the Onset Delay was 100 ms. However, a consideration of the pattern of results suggest that the effect of increased variance did not substantially affect the pattern of results found in this experiment.

**Figure 7:**
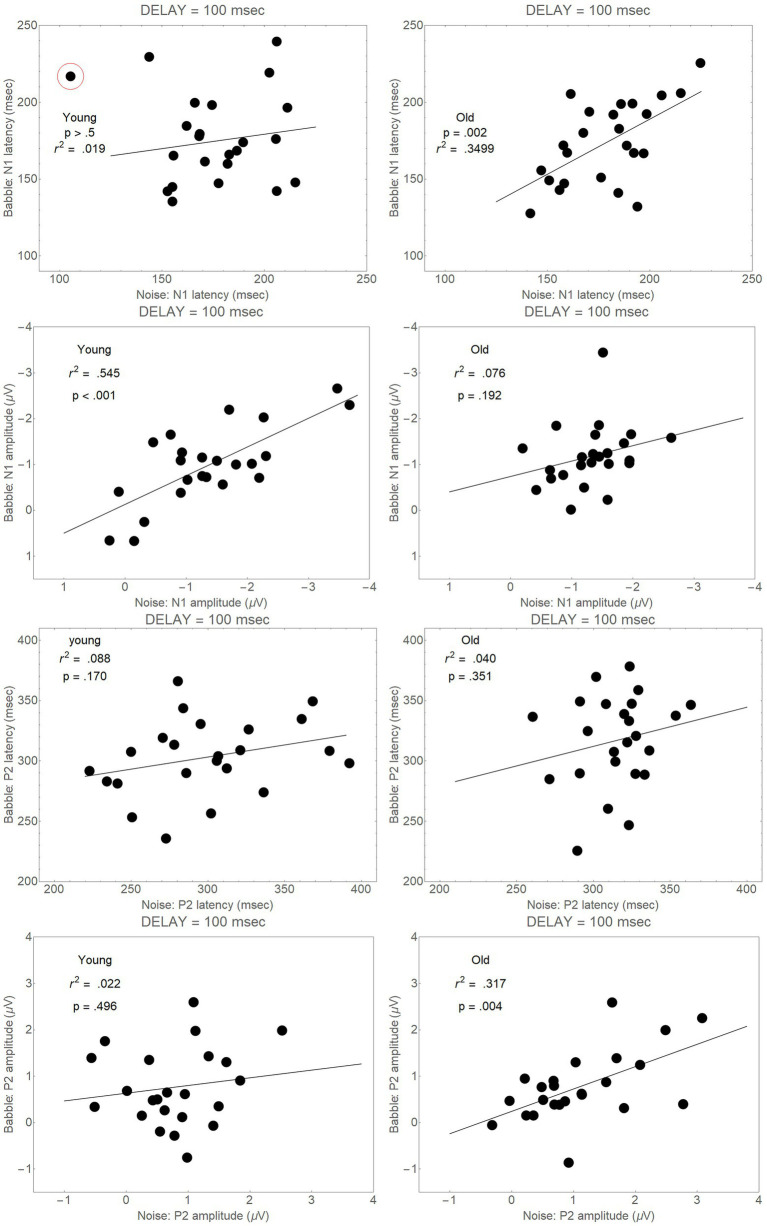
Correlations between Noise and Babble latencies and between Noise and Babble peaks for Onset Delay = 100 ms for both N1 and P2 peaks. The circled point in the top left-hand panel was not included when the correlations were conducted because it was too close to the lower boundary for latency and the upper boundary for amplitude.

First, if the failure to find differences among the conditions in the Onset Delay = 100 ms analyses were due to noisiness in the ERP difference wave, we would not expect to find correlations between Babble and Noise latencies, or between Babble and Noise peaks. Figure 7, however, shows that positive correlations are found in both younger and older adults between Noise and Babble latencies and Noise and Babble peaks for both age groups for both N1 and P2 waveforms. Moreover, three of these correlations were highly significant (*p* < 0.005 for all three correlations), while a fourth was marginally significant (*p* = 0.076). If the difference waveform was highly variable, we would not expect to find such correlations between Noise and Babble latencies and between Noise and Babble peak amplitudes.

Second, it is unlikely that we would find, as predicted by the Ben-David et al. (2012) study, that the CI rectangles for the Babble masker occupied the same position in the two-dimensional CI space for Onset Delays of 100 and 600 ms for both N1 and P2 waves (see Figure 5).

Third, it also unlikely that we would find, as predicted by the Ben-David et al. (2012) study, that the CI rectangles for Noise and Babble in younger adults would overlap for Onset Delays of 600 ms for both N1 and P2 waveforms (see Figure 6, panels D and H), but not as much for older adults in the same two conditions (Figure 6, panels C and G).

These three factors make it less likely that the failure to find differences among the four groups for an Onset Delay of 100 ms for both N1 and P2 waves is simply due to an increased variability in the difference wave that is used to determine N1 and P2 peak latencies and peak amplitude for an Onset Delay = 100 ms.

## Conclusion

The N1 and P2 waves in the ACC are thought to be associated with early acoustical processing of the auditory target in a noisy background. The Ben-David et al. (2012) results indicate that increasing the Onset Delay between the masker and the target word from 100 to 600 ms results in a release from masking in both Noise and Babble for young adults, but only in Noise for older adults. If the release from masking due to Onset Delay occurs in the early stages of cortical processing, then we would expect N1 and P2 waves to be similar for both younger and older adults when the Onset Delay was too short (100 ms) to produce a release from masking—provided that the SNR was adjusted to produce equivalent per cent correct word identification in both younger and older adults, as they were in all of the conditions of this experiment. The results of this experiment confirmed this prediction. If a longer Onset Delay (600 ms) resulted in a release from masking for younger and older adults in Noise, but only for younger adults in Babble, and if the release from masking occurred in the early stages of cortical processing of the target word, we would expect to see evidence for this in both the N1 and P2 waves. This prediction was also confirmed in that we found both the N1 and P2 waveforms of older adults in Babble did not change when the Onset Delay was increased from 100 to 600 ms (see Figure 5). In addition, for younger adults, the two waveforms for Noise and Babble maskers were same when the Onset Delay was 600 ms, whereas they were different in older adults (see Figure 6). We would expect the waveforms for Babble and Noise to be the same in younger adults if they were experiencing the same amount of release from masking at this delay. Conversely, in older adults, we would expect the waveforms to be different at this delay if they were experiencing a release from masking in Noise but not in Babble. Finally, we would expect the waveforms in those conditions in which there was a release from masking at an Onset Delay of 600 ms (Young-Noise, Young-Babble, and Old-Noise) to be distinct (have different latencies and peak amplitude) from those in which there was no release from masking (Old Babble). Figure 4 (right panel) and Table 2 indicate that the results of this experiment support this prediction. Hence, the electrophysiological results strongly indicate that the automatic release from masking (the segregation of the target word from the masker background) due to an Onset-Delay between Masker and Word Target, occurs in the early stages of acoustic processing of the word.

Because this release from speech masking takes place in the early stages of cortical processing and is likely to be automatic, the only remedy available to health-care practitioners to help older adults compensate for this specific age-related deficit is to improve the SNR. This can be accomplished by (1) manipulating the acoustic scene to shield older adults from competing speech, and/or (2) using directional microphones and/or noise reduction technology to improve the SNR. Otherwise, older adults are likely to continue struggling with speech recognition in the presence of multiple competing talkers, in part, because of their limited ability to use temporal onset cues to facilitate stream segregation so that they can more readily focus their attention on processing the targeted speech.

## Data Availability Statement

The raw data supporting the conclusions of this article will be made available by the authors, without undue reservation.

## Ethics Statement

The studies involving human participants were reviewed and approved by Office of Research Ethics (ORE) University of Toronto, Mississauga. The patients/participants provided their written informed consent to participate in this study.

## Author Contributions

BS, MA-R, and DK conceived and planned the experiments. CR, DK, and MA-R carried out the experiments. SA was responsible for setting up and calibrating the EEG apparatus. CA and CR processed the EEG recordings. BS took the lead in statistically analysing the data. BS, CR, MA-R, and CA contributed to the writing of the manuscript. All authors contributed to the article and approved the submitted version.

## Funding

This research was supported by a grant from the Natural Sciences and Engineering Research Council of Canada (ROPIN9952-13 and RGPIN-2021-02721).

## Conflict of Interest

The authors declare that the research was conducted in the absence of any commercial or financial relationships that could be construed as a potential conflict of interest.

## Publisher’s Note

All claims expressed in this article are solely those of the authors and do not necessarily represent those of their affiliated organizations, or those of the publisher, the editors and the reviewers. Any product that may be evaluated in this article, or claim that may be made by its manufacturer, is not guaranteed or endorsed by the publisher.
