# Supplementary material for: Age-Related Differences in Early Cortical Representations of Target Speech Masked by Either Steady-State Noise or Competing Speech
Source: Front Psychol. 2022 Aug 4;13:935475. doi: 10.3389/fpsyg.2022.935475 (PMC9389464; doi:10.3389/fpsyg.2022.935475)
Supplement: Supplementary file 1 [file Table_1.pdf]

Appendix: Statistical Analyses

We first assume that the x and y measures have a bi-normal distribution in each of the two groups (referred to here as groups A and B, respectively). We then evaluate the null hypothesis that the bi-normal distribution of the x and y measures in group B is the same as it is in group A. This evaluation is carried out using confidence intervals for the x and y measures in the two groups. We start by defining 95% confidence limits for the x and y measures in the two groups. Let $\mu_{A,x}$ and $\sigma_{A,x}$ represent the population mean and standard deviation of the x measures in Group A, and $\mu_{B,y}$ and $\sigma_{B,y}$ represent the population mean and standard deviation of the y measures in Group B. The estimates of $\mu_{A,x}, \mu_{B,x}, \mu_{A,y}, \mu_{B,y}$ are $\bar{x}_A, \bar{x}_B, \bar{y}_A, \bar{y}_B$, respectively. The estimates of $\sigma_{A,x}, \sigma_{B,x}, \sigma_{A,y}, \sigma_{B,y}$ are $s_{A,x}, s_{B,x}, s_{A,y}, s_{B,y}$, respectively. This yields a total of four confidence intervals: (a) a 95% confidence interval for $\mu_{A,x}$, (b) a 95% confidence interval for $\mu_{A,y}$,(c) a 95% confidence interval for $\mu_{B,x}$, and (d) a 95% confidence interval for $\mu_{B,y}$. These four confidence intervals are then used to define two rectangles in a two-dimensional plot. The lengths of the lower and upper horizontal lines of the rectangle representing group A are given by the difference between the upper and lower boundaries of the confidence interval for $\mu_{A,x}$. The lengths of the left and right vertical lines of the rectangle representing group A are given by the difference between the upper and lower boundaries of the confidence interval for $\mu_{A,y}$. The rectangle for group A is centred at $\{\bar{x}_A, \bar{y}_A\}$, where $\bar{x}_A$ is the sample mean of the x measures in Group A, and $\bar{y}_A$ is the sample mean of the y measures in group A. The same procedure is used to construct the rectangle representing Group B. Figure A1 shows two such rectangles based on the following confidence intervals for $n_A = 24$ individuals in Group A and $n_B = 23$ individuals in group B. For 24 hypothetical individuals in Group A,

$$Group\ A\!: n_A = 24, \bar{x}_A = 145, s_{A,x} = 35,\ \bar{y}_A = 0.8,\ s_{A,y} = 0.2$$

$$CI[\mu_{A,x}]\!: LB_{\mu_{A,x}} = 145 - |t_{.025,df=23}| * \frac{35}{\sqrt{24}} = 130.221, UB_{\mu_{A,x}} = 145 + |t_{.025,df=23}| * \frac{35}{\sqrt{24}} = 159.779$$

$$CI[\mu_{A,y}]\!: LB_{\mu_{A,y}} = 0.8 - |t_{.025,df=23}| * \frac{.2}{\sqrt{24}} = .715547, UB_{\mu_{A,y}} = .8 + |t_{.025,df=22}| * \frac{.2}{\sqrt{24}} = .884453$$

For 23 hypothetical individuals in Group B,

$$Group\ B\!: n_B = 23, \bar{x}_B = 152, s_{B,x} = 31, \bar{y}_B = 0.65, s_{B,y} = 0.25$$

$$CI[\mu_{B,x}]\!: LB_{\mu_{B,x}} = 152 - |t_{.025,df=22}| * \frac{31}{\sqrt{23}} = 138.595, UB_{\mu_{B,x}} = 152 + |t_{.025,df=22}| * \frac{31}{\sqrt{23}} = 165.405$$

$$CI[\mu_{B,y}]\!: LB_{\mu_{B,y}} = 0.65 - |t_{.025,df=22}| * \frac{.25}{\sqrt{23}} = .541892\ , UB_{\mu_{B,y}} = .65 + |t_{.025,df=22}| * \frac{.25}{\sqrt{23}} = .758108$$

In Figure A1, the rectangle associated with group A is shown in red, whereas the rectangle associated with group B is shown in green. Note that the two rectangles overlap. We can label the overlapping portion of the two rectangles A and B as rectangle AB. The rectangle defined by this overlap and the associated confidence intervals can be used to determine the probability of observing that both of the population means for Group A $\{\mu_{A,x}, \mu_{A,y}\}$ fall into this overlap rectangle. There is a 95% probability that $\mu_{A,x}$ is located somewhere within the red rectangle, and a 95% probability that $\mu_{A,y,}$ is also located somewhere within the red rectangle. Hence, there is an 90.25% chance that both of these population means fall somewhere within the red rectangle. We will use these four confidence intervals to determine the probability that both $\mu_{A,x}$ and $\mu_{A,y}$ are simultaneously located in the region where the red rectangle overlays the green rectangle.

Figure A1. An example of an overlap between two rectangles: A (red), and B (green).

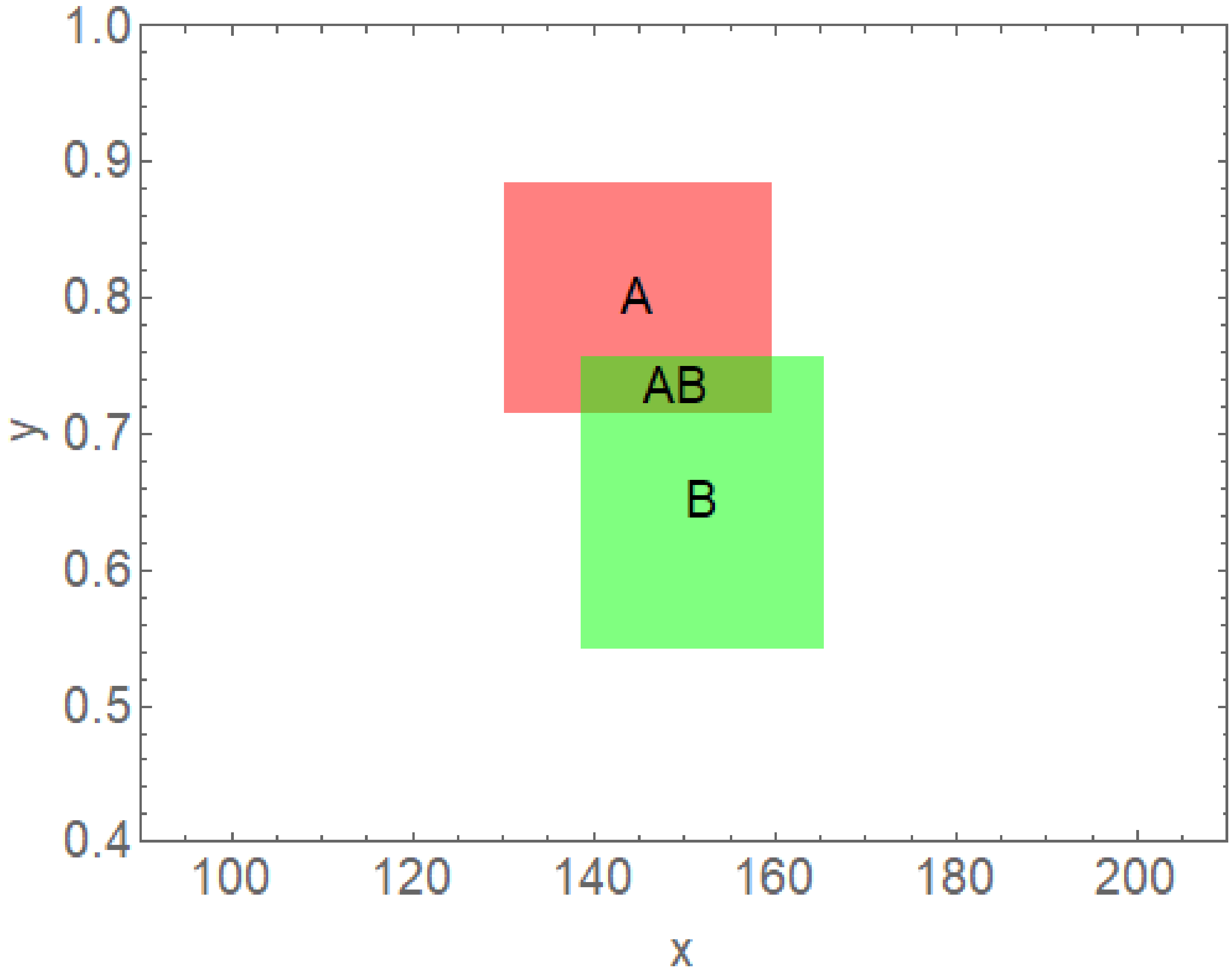

To see how to do this, we first convert the lower and upper boundaries of the confidence interval for $\mu_{A,x}$ into t-scores according to the formulae below to obtain

$$t_{LB_{A,x}} = \frac{LB_{A,x} - \bar{x}_A}{\frac{s_{A,x}}{n_A}} = \frac{130.221-145}{\frac{35}{\sqrt{24}}} = -2.06866\,, t_{UB_{A,x}} = \frac{UB_{A,x} - \bar{x}_A}{\frac{s_{A,x}}{\sqrt{n_A}}} = \frac{159.779-145}{\frac{35}{\sqrt{24}}} = 2.06866,$$

Note that $t_{LB_{A,x}}$ and $t_{UB_{A,x}}$ are the .025 and .975 points on the density function for Student's t distribution with 23 degrees of freedom. Hence, the integral of this density function between these two limits is, of course, equal to 0.95. We can now use this density function to find the probability that both $\mu_{A,x}$ *and* $\mu_{A,y}$ are found in rectangle AB.

To compute the probability that $\mu_{A,x}$ is to be found within rectangle AB, we need to express the lower x boundary for group B (which is the lower boundary of the rectangle AB) in terms of the t-distribution for Group A. Note that upper boundary of the rectangle AB is already specified in with respect to the t-distribution for group A $\left(t_{UB_{A,x}} = 2.06866\right)$. In general, we can translate both the lower and upper boundaries of the confidence interval for x in Group B, into a t-scores with respect to the t-distribution for the confidence interval for x in Group A. To see this, note that the 95% confidence interval on the location of the mean latency in Group A specifies that

$$p\left[\bar{x}_A - \left|t_{.025,df=n_A-1}\right| * \frac{s_{x_A}}{\sqrt{n_A}} < \mu_{A,x} < \bar{x}_A + \left|t_{.025,df=n_A-1}\right| * \frac{s_{x_A}}{\sqrt{n_A}}\right] = 0.95$$

Now we can shrink the size of the confidence interval for $\mu_{A,x}$ by using a larger value of α. This smaller confidence interval is

$$p\left[\bar{x}_A - \left|t_{\frac{\alpha}{2},df=n_A-1}\right| * \frac{s_{x_A}}{\sqrt{n_A}} < \mu_{A,x} < \bar{x}_A + \left|t_{\frac{\alpha}{2},df=n_A-1}\right| * \frac{s_{x_A}}{\sqrt{n_A}}\right] = 1-\alpha$$

Now suppose the upper boundary of the shrunken confidence interval is set to the upper boundary of the 95% confidence limit for group B ($UB_{B,x}$). Then we have

$$UB_{B,x} = \bar{x}_A + \left|t_{\frac{\alpha}{2},df=n_A-1}\right| * \frac{s_{x_A}}{\sqrt{n_A}}$$

Now we can solve this equation for $\left|t_{\frac{\alpha}{2},df=n_A-1}\right|$ which yields

$$\left|t_{\frac{\alpha}{2},df=n_A-1}\right| = \frac{UB_{B,x} - \bar{x}_A}{\frac{s_{x_A}}{\sqrt{n_A}}} = t[UB_{B,x} in\ A]$$

Hence, $\frac{UB_{B,x}-\bar{x}_A}{\frac{s_{x_A}}{\sqrt{n_A}}}$ is the t value corresponding to the upper boundary for the confidence interval for Group B expressed in terms of the confidence interval for A, and its t value is $\left|t_{\frac{\alpha}{2},df=n_A-1}\right|$. If we now set the lower boundary of the 95% confidence limit for Group B ($LB_{B,x}$) relative to Group A's confidence interval, we have

$$LB_{B,x} = \bar{x}_A - \left|t_{\frac{\alpha}{2},df=n_A-1}\right| * \frac{s_{x_A}}{\sqrt{n_A}}$$

If we solve this equation for $-\left|t_{\frac{\alpha}{2},df=n_A-1}\right|$ we have

$$-\left|t_{\frac{\alpha}{2},df=n_A-1}\right| = \frac{LB_{B,x} - \bar{x}_A}{\frac{s_{x_A}}{\sqrt{n_A}}} = t[LB_{B,x} in\ A]$$

Hence, $\frac{LB_{B,x}-\bar{x}_A}{\frac{s_{x_A}}{\sqrt{n_A}}}$ is the t value of the lower boundary for the confidence interval for Group B expressed in terms of the confidence interval for A ($t[LB_{B,x} in\ A]$), and its t value is - $\left|t_{\frac{\alpha}{2},df=n_A-1}\right|$. In this way we can determine the t-score equivalents of the lower and upper boundaries of Group B in terms of their t-values referenced to Group A, and vice versa.

With respect to Figure A1, the t value of the lower boundary of the confidence interval for Group B expressed in A is

$$t[LB_{B,x} in\ A] = \frac{LB_{B,x} - \overline{x}_A}{\frac{s_{A,x}}{\sqrt{n_A}}} = \frac{138.595 - 145}{\frac{35}{\sqrt{24}}} = -.89657$$

The upper boundary of the confidence interval for x in Group A is

$$t_{UB_{A,x}} = \frac{UB_{A,x} - \overline{x}_A}{\frac{s_{A,x}}{\sqrt{n_A}}} = \frac{159.779 - 145}{\frac{35}{\sqrt{24}}} = 2.06866$$

Hence, the probability of $\mu_{A,x}$ being in within the rectangle AB is equal to the integral of Student's t-distribution (df =23) from $t[LB_{B,x} in\ A]$ to $t_{UB_{A,x}}$, i.e.,

$$p\left[\, t[LB_{B,x} in\ A] \ < \ \mu_{A,x} < t_{UB_{A,x}} \right] = \int_{t=\ t[LB_{B,x} in\ A]}^{t=t_{UB_{A,x}}} D(t)dt = \ .785381 \ \ (for\ df\ = n_A - 1 = 23)$$

where D(t) is the probability density function for Student's t with 23 degrees of freedom. By a similar argument, the probability of $\mu_{A,y}$ being in the rectangle AB is obtained by determining the t value of the lower boundary of the confidence interval for $\mu_{A,y}$ and expressing the upper boundary of the confidence interval on $\mu_{B,y}$ in terms of the rectangle A, i.e.

$$t_{LB_{A,y}} = \frac{LB_{A,y} - \overline{y}_A}{\frac{s_{A,y}}{\sqrt{n_A}}} = \frac{.715547 - .8}{\frac{.2}{\sqrt{24}}} = -2.06866, t[UB_{B,y} in\ A] \ = \frac{UB_{B,y} \ - \overline{y}_A}{\frac{s_{A,y}}{\sqrt{n_A}}} = \frac{.758108 - .8}{\frac{.2}{\sqrt{24}}} = -1.02614$$

It follows that the probability of $\mu_{A,y}$ being in within the rectangle AB is equal to the integral of Student's t-distribution (df =23) from $t_{LB_{A,y}}$ to $t[UB_{B,y} in\ A]$, i.e.,

$$p\left[t_{LB_{A,y}} \ < \ \mu_{A,y} < t[UB_{B,y} in\ A] \,\right] = \int_{t=t_{LB_{A,y}}}^{t=\ t[UB_{B,y} in\ A]} D(t)dt = \ .132749 \ \ (for\ df\ = n_A - 1 = 23)$$

Therefore the probability that both $\mu_{A,x}$ and $\mu_{A,y}$ are rectangle AB is .785381*.132749 = .104259.

To find the probability that both $\mu_{B,x}$ and $\mu_{B,y}$ are to be found in rectangle AB, we follow a similar procedure. We find the t-score corresponding to the lower boundary of the confidence interval for $\mu_{B,x}$, the t-score of the upper boundary of the confidence interval for $\mu_{A,x}$ expressed in terms of the rectangle B, the t-score corresponding to the lower boundary of the confidence interval for $\mu_{A,y}$ expressed in terms of rectangle B, and the t-score corresponding to the upper boundary of the confidence interval for $\mu_{B,y}$. These four t values for determining the probability of finding $\mu_{B,x}$ and $\mu_{B,y}$ in the rectangle AB are:

$$t_{LB_{B,x}} = \frac{LB_{B,x} - \overline{x}_B}{\frac{s_{B,x}}{\sqrt{n_B}}} = \frac{138.595 - 152}{\frac{31}{\sqrt{23}}} = -2.07387 \ , t[UB_{A,x} in\ B] = \frac{UB_{A,x} - \overline{x}_B}{\frac{s_{B,x}}{\sqrt{n_B}}} = \frac{159.779 - 152}{\frac{31}{\sqrt{23}}} = 1.20348$$

$$t[LB_{A,y} in\ B] \ = \frac{LB_{A,y} - \overline{y}_B}{\frac{s_{B,y}}{\sqrt{n_B}}} = \frac{.715547 - .65}{\frac{.25}{\sqrt{23}}} = 1.25741, t_{UB_{B,y}} = \frac{UB_{B,y} - \overline{y}_B}{\frac{s_{B,y}}{\sqrt{n_B}}} = \frac{.758108 - .65}{\frac{.25}{\sqrt{23}}} = 2.07387,$$

$$p\left[t_{LB_{B,x}} \ < \ \mu_{B,x} < \ t[UB_{A,x} in\ B] \,\right] = \int_{t=t_{LB_{B,x}}}^{t=t[UB_{A,x} in\ B]} D(t)dt = \ .854209 \ \ (for\ df\ = n_B - 1 = 22)$$

$$p\left[\ t\left[LB_{A,y} in\ B\right] < \ \mu_{B,y} < t_{UB_{B,y}} \right] = \int_{t=t[LB_{A,y} in\ B]}^{t=t_{UB_{B,y}}} D(t)dt = \ .085949 \ \ (for\ df\ = n_B - 1 = 22)$$

Hence, the probability of both $\mu_{B,x}$ and $\mu_{B,y}$ being in rectangle AB is .854209*.0858949 = .073372.

These calculations show that we can compute the probability that both $\mu_{A,x}$ and $\mu_{A,y}$ are in rectangle AB, as well as the probability that both $\mu_{B,x}$ and $\mu_{B,y}$ are in in rectangle AB, for this particular type of intersection between triangles A and B (lower right hand corner of A intersecting the upper left-hand corner of B). However, there are a total of 16 different ways two rectangles can overlap with one another as shown in Figure A2. Each of these different configurations of overlap require different limits of integration in order to determine the probability that both $\mu_{A,x}$ and $\mu_{A,y}$ are in rectangle AB, as well as the probability that both $\mu_{B,x}$ and $\mu_{B,y}$ are in in rectangle AB. I have written a program that takes all of these 16 patterns of interaction into account. In addition, there is the possibility that there is

no overlap at all. In that event, the probability that both $\mu_{A,x}$ and $\mu_{A,y}$ are in rectangle AB, as well as the probability that $\mu_{B,x}$ and $\mu_{B,y}$ are in in rectangle AB are both 0. Hence, we can always determine the probability that both $\mu_{A,x}$ and $\mu_{A,y}$ are in rectangle AB, as well as the probability that both $\mu_{B,x}$ and $\mu_{B,y}$ are in in rectangle AB.

Figure A2. All possible ways in which a green rectangle can overlap with a red rectangle.

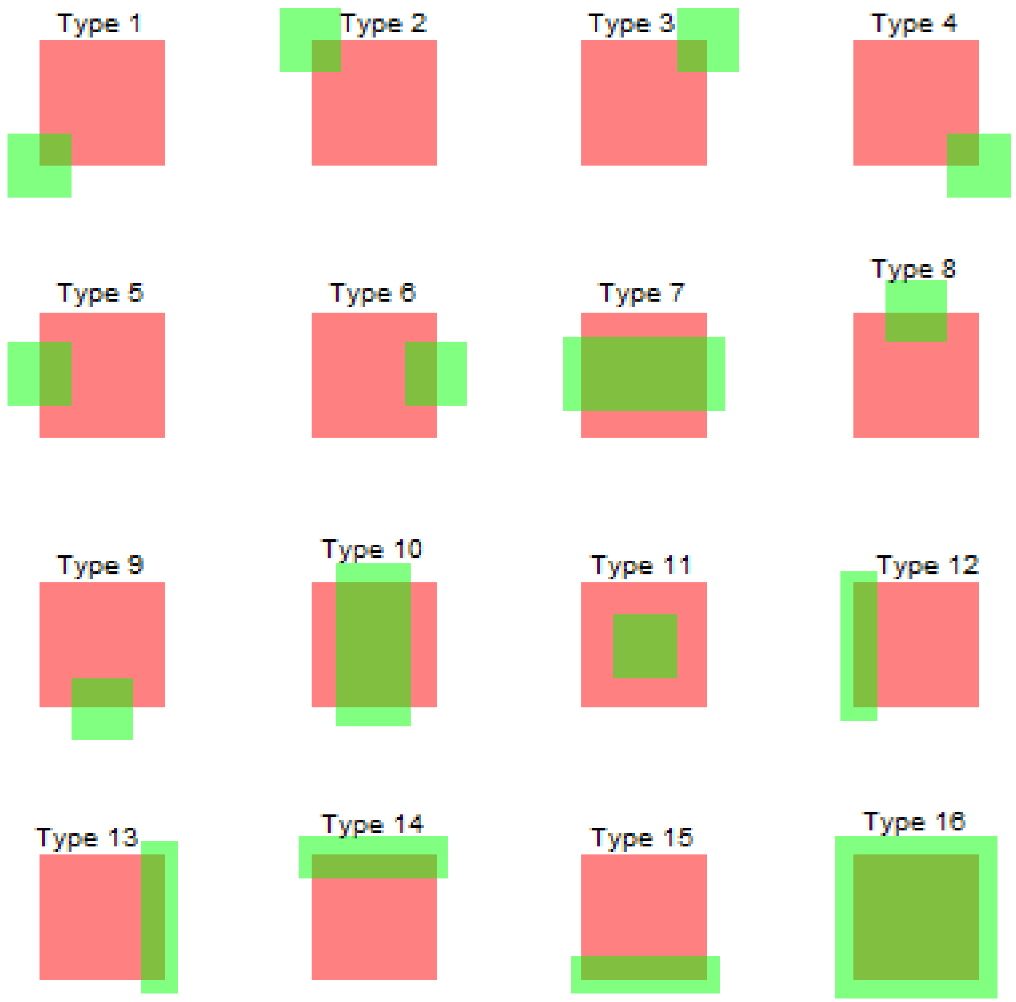

Note that the model allows $\mu_{A,x}$ to differ from $\mu_{B,x}$ and $\mu_{A,y}$ to differ from $\mu_{B,y}$. We will now evaluate what we would expect if the two groups had the same population means for the x and y variables. If this null hypothesis were true, we would expect a greater degree of overlap between the green and red rectangles. If we could derive the probability density functions for the population means from the red group falling into the overlap between the red and green groups, as well as the probability density functions for the population means from the green group falling into the overlap between red and green groups, we could test the null hypothesis that both groups had identical population means for the x and y variables. Unfortunately, I have been unable to do this analytically. However, these probability density functions were investigated using Monte Carlo techniques. This permits us to test null hypotheses for the overlap between any two confidence interval rectangles. Before we do so, there are a couple of issues that we should address.

The first is to determine the extent to which a correlation between the x and y variables could affect the probability of the population means falling the overlap area. This concern can be readily dismissed. The probability density functions for the x and y variables considered separately are unaffected by the correlation between them. The second concern is the extent to which differences in population means for x and y could affect the probability of both of

them falling into the overlap region. This concern also is not an issue since the analysis conducted here is based on the confidence intervals for the two means, and as such they will not be affected by the particular values of the means.

A third issue that is not so easily dismissed is the extent to which the coefficient of variation (the ratio of the standard deviation to the mean) might affect the probability densities used in evaluating the null hypotheses. To determine the extent to which the coefficient of variation might affect the density functions associated with the intersection of the red and green rectangles when the null hypothesis is true (both groups have the same population means), we generated 500,000 samples under this null hypothesis, and computed two test statistics (the probability that the population means of the red group fell into the intersection of the red and green groups, and the probability that the population means of the green group fell into the intersection of the red and green groups. If the research hypotheses were true, then we would expect lower probability estimates in these two cases. Hence, we determined the estimates corresponding to lower 2.5 , 5, 10, and 50th percentiles of these distributions, with the 50th percentile being the median value. Twenty-five such simulations were conducted, completely crossing five coefficients of variation (.05, .1, .2, .3, .4) used for the x variable, with the same 5 coefficients of variation used for the y variable. Table A1 presents the critical test statistic values for the different combinations of coefficients of variation for both groups A and B. There is very little indication of any significant variation in the critical values of the test statistics among the 25 combination of coefficients of variation over a 16 to 1 range. Critical values of the test statistics are slightly lower for group B whose confidence intervals were based on a fewer degrees of freedom (df=22) than that in Group A (df =23). Hence, as expected, the critical values required for significance drop slightly as the degrees of freedom decrease. Given the results of these simulations we required a value of the test statistic for Group A to be < .143 to reach significance at the .10 level, < .063 to reach significance at the .05 level, and < .021 for significance at the .025 level. For group B we required the test statistic to be < .135 for significance at the .10 level, < .059 for significance at the .05 level, and < .020 for significance at the .025 level.

Table A1. Groups A and B simulation results. Cell entries under the null hypothesis are: the median, followed by significance levels for $\alpha = .10$, $\alpha = .05$ and $\alpha = .025$ (1-tail test). Rows specify the coefficients of variation used for μ_x. Columns specify the coefficients of variation used for μ_y. There were 24 and 23 participants in Groups A and B, respectively. Coefficients of variation ranged from .05 to .8 for both variables. The number of simulations was 500,000.

Group A, n = 24				(μ_y)		
	Coefficient of Variation	.05	.1	.2	.4	.8
	.05	.597<br>.145<br>.063<br>.021	.595<br>.145<br>.064<br>.022	.597<br>.145<br>.063<br>.021	.597<br>.144<br>.063<br>.021	.596<br>.145<br>.064<br>.022
	.1	.596<br>.144<br>.063<br>.021	.596<br>.145<br>.063<br>.021	.596<br>.144<br>.063<br>.022	.596<br>.145<br>.063<br>.022	.596<br>.143<br>.063<br>.021
(μ_x)	.2	.596<br>.145<br>.064<br>.022	.596<br>.144<br>.063<br>.021	.596<br>.145<br>.063<br>.021	.596<br>.144<br>.062<br>.021	.596<br>.145<br>.063<br>.022
	.4	.596<br>.145<br>.063<br>.022	.597<br>.145<br>.063<br>.021	.597<br>.145<br>.064<br>.022	.597<br>.145<br>.063<br>.021	.596<br>.144<br>.062<br>.021
	.8	.597<br>.145<br>.064<br>.022	.595<br>.144<br>.063<br>.021	.596<br>.143<br>.063<br>.021	.596<br>.144<br>.064<br>.022	.595<br>.143<br>.063<br>.022

Group B, n = 23				(μ_y)		
	Coefficient of Variation	.05	.1	.2	.4	.8
	.05	.572<br>.136<br>.059<br>.020	.570<br>.136<br>.060<br>.021	.571<br>.136<br>.060<br>.020	.571<br>.136<br>.060<br>.020	.572<br>.137<br>.060<br>.020
	.1	.571<br>.135<br>.059<br>.020	.571<br>.136<br>.060<br>.020	.571<br>.137<br>.060<br>.020	.571<br>.136<br>.060<br>.021	.571<br>.136<br>.060<br>.020
(μ_x)	.2	.571<br>.136<br>.060<br>.021	.570<br>.136<br>.059<br>.020	.571<br>.136<br>.059<br>.021	.571<br>.135<br>.059<br>.020	.571<br>.137<br>.060<br>.020
	.4	.571<br>.136<br>.060<br>.021	.571<br>.136<br>.059<br>.020	.572<br>.137<br>.060<br>.021	.571<br>.136<br>.059<br>.020	.571<br>.135<br>.059<br>.020
	.8	.572<br>.137<br>.060<br>.021	.570<br>.136<br>.059<br>.020	.571<br>.135<br>.059<br>.020	.571<br>.136<br>.060<br>.021	.570<br>.135<br>.060<br>.021
